# Face-to-face intubation using a lightwand in a patient with severe thoracolumbar kyphosis: a case report

**DOI:** 10.1186/s12871-018-0556-y

**Published:** 2018-07-21

**Authors:** Hyungmo Jeong, Minchul Chae, Hyungseok Seo, Jae-Woo Yi, Jong-Man Kang, Bong-Jae Lee

**Affiliations:** Department of Anesthesiology and Pain Medicine, Kyung Hee University Hospital at Gangdong, College of Medicine, Kyung Hee University, 892 Dongnam-ro Gangdong-gu, Seoul, 05278 South Korea

**Keywords:** Face-to-face intubation, Lightwand, Difficult airway, Kyphosis

## Abstract

**Background:**

Severe deformity of the thoracolumbar spine may cause difficulty in airway management during induction of anesthesia. Therefore, special attention must be devoted to patient safety.

**Case presentation:**

A 65-year-old male with severe thoracolumbar kyphosis was scheduled to undergo posterior spinal fusion under general anesthesia. Due to his inability to lie supine, conventional tracheal intubation under direct laryngoscopy was difficult. Alternatively, face-to-face tracheal intubation using a lightwand in the semi-recumbent position was performed. Intubation was successful on the first attempt without any complications.

**Conclusions:**

The face-to-face intubation technique using a lightwand is one of several alternative techniques for tracheal intubation in patients who cannot lie supine.

## Background

Severe spinal deformities, including thoracolumbar kyphosis, can cause difficulty in airway management; therefore, special focus on a successful airway management strategy is critical to ensure patient safety. As most patients with severe kyphosis cannot be appropriately placed in the supine position, there can be significant difficulty in performing direct or video laryngoscopy; consequently, an alternative intubation technique is required [1]. The face-to-face intubation technique is such an alternative, and may provide an appropriate approach in patients in a sitting position [2]. The lightwand is a lighted stylet that uses the principle of transillumination of the soft tissues of the anterior neck to guide the tracheal tube into the trachea. Thus, it may cause less dental trauma, mucosal injury, and hemodynamic instability compared with direct laryngoscopy [3]. Herein, we report a successful face-to-face intubation using a lightwand in a patient with severe thoracolumbar kyphosis.

## Case presentation

**Fig. 1:**
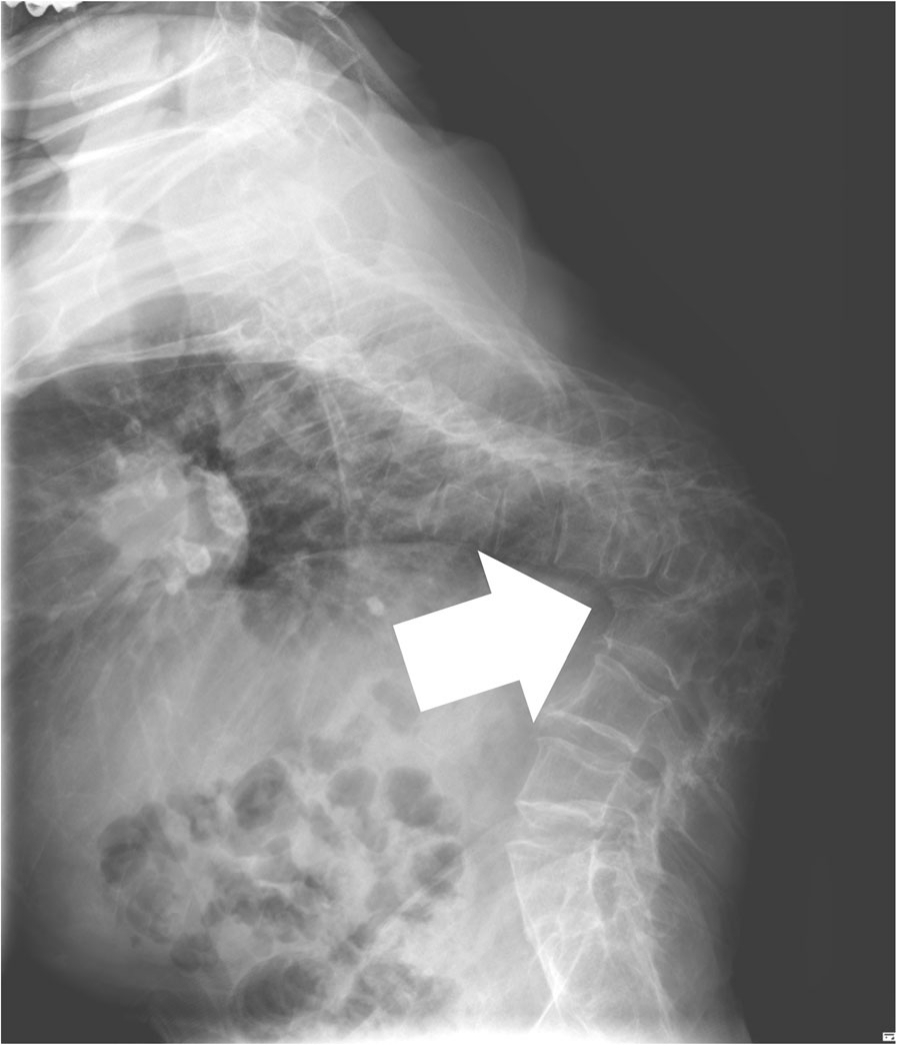
Thoraco-lumbar kyphosis. The patient exhibited severe kyphosis (approximately 115 degrees), with abnormal contour changes and vertebral body fusions at the level of T10–L3 (white arrow)

A 65-year-old male, 155 cm tall and weighing 53 kg, was scheduled to undergo mesh cage insertion and posterior spinal fusion from T6 to L5 for severe kyphosis due to spinal tuberculosis. Preoperative chest radiography revealed severe kyphosis of the thoracolumbar spine; however, there was no active lesion in the lungs. Thoracolumbar magnetic resonance imaging and computed tomography revealed spinal fusion at the level of T9–L3, with volume decrease and deformity associated with severe kyphosis. The kyphotic angle was approximately 115 degrees (Fig. 1). Preoperative pulmonary function tests revealed a mild restrictive pattern with a forced vital capacity (FVC) of 1.81 L (63% of normal), a forced expiratory volume in 1 s (FEV_1_) of 1.53 L (73% of normal), and an FEV_1_/FVC ratio of 85%. Preoperative electrocardiography revealed normal sinus rhythm. In the preoperative visit, the patient exhibited limited neck motion because of severe kyphosis; he was Mallampati class III.

**Fig. 2:**
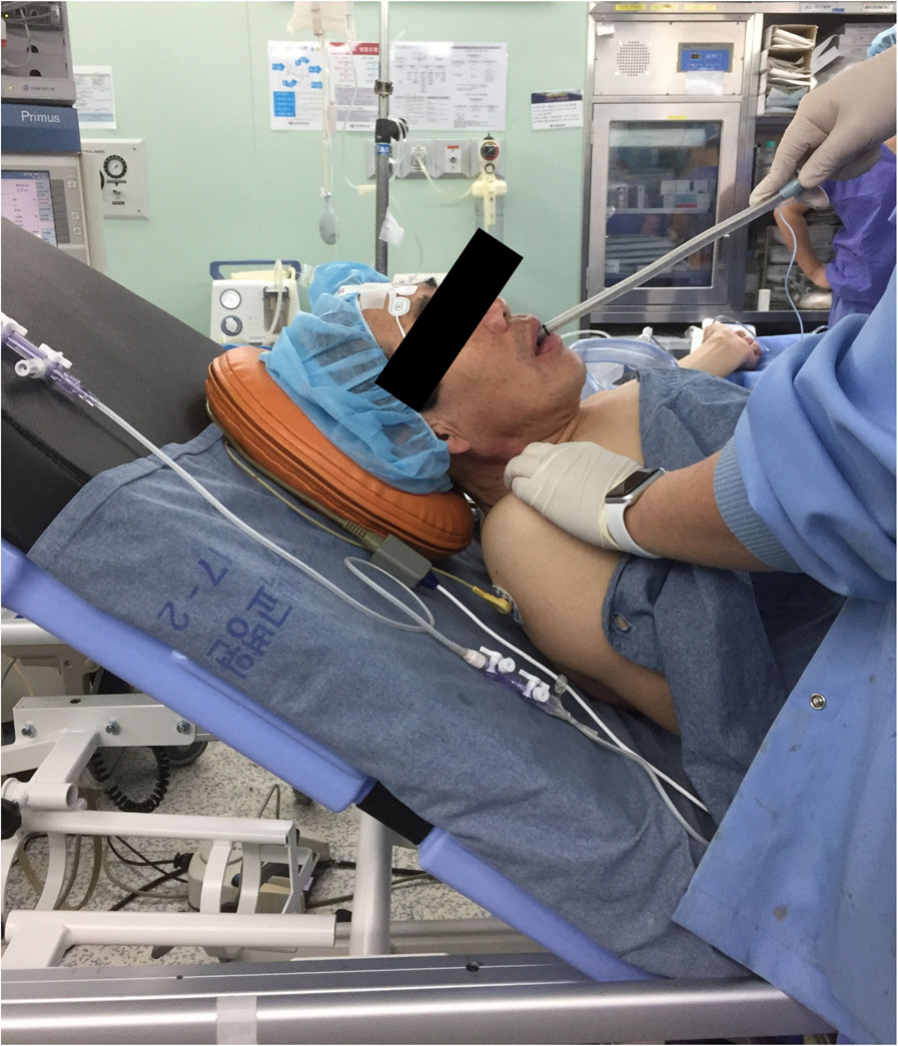
Position during tracheal intubation. Face-to-face intubation using a lightwand was performed successfully with the patient in the sitting position

In the operating room, the patient was monitored using three-lead electrocardiography, pulse oximetry, non-invasive blood pressure monitoring, and bispectral index. Due to inability to lie on his back without any supportive devices, the head of the operating bed was raised approximately 30–40 degrees, and anesthesia was induced in the semi-recumbent position. Anesthesia was induced using a target-controlled infusion of propofol (Schnider model) and remifentanil (Minto model); rocuronium bromide 0.6 mg/kg was administered to facilitate tracheal intubation. Before tracheal intubation, preoxygenation was performed for 5 to 10 min with 100% oxygen in a face-to-face approach, with bimanual mask holding and mechanical ventilation. Although the awake intubation technique may be considered in cases of anticipated difficult intubation, the cause of difficult intubation in the present case was attributed to a kyphotic change, and not the airway itself, such as an anteriorly deviated glottis or soft tissue swelling; therefore, the authors believed that the patient’s airway could be adequately maintained using bimanual mask-holding in a face-to-face position. Initially, direct laryngoscopy and video laryngoscopy were attempted; however, it was exceedingly difficult to visualize the vocal cords because of the patient’s semi-recumbent position, his short stature, and the relatively large bed size. Moreover, a conventional lightwand technique from the head end of the patient was also difficult to perform. Although fiberoptic bronchoscopy can be considered as a first choice, it was temporarily unavailable because of an insufficient number of devices and well-trained staff. As the conventional overhead approach using laryngoscopy or lightwand was difficult, an alternative face-to-face approach using a lightwand was attempted. After all lights in the operating room were turned off, the anesthetist opened the patient’s mouth while facing him, and slowly inserted the tracheal tube-launched lightwand, of which the tip was bent at an angle of 90 degrees along the base of the tongue in the midline. The bright red light at the tip of the stylet was transilluminated and positioned at the center of the neck; subsequently, the tracheal tube was gently inserted at the location of light on the neck. (Fig. 2). Face-to-face lightwand intubation was successful on the first attempt, and no specific complications, such as hemodynamic instabilities, intraoral traumas, or others, were encountered. After tracheal intubation, invasive arterial blood pressure was monitored through a cannula placed in the left radial artery using a hemodynamic monitoring device (EV1000 clinical platform, Edward Lifesciences Corp., Irwin, CA, USA). The surgery lasted approximately 7 h, during which vital signs were adequately maintained. After completion of surgery, neuromuscular blockade was reversed using sugammadex 200 mg followed by extubation in the operation room. Adequacy of respiration was confirmed, and the patient was transferred to the intensive care unit with continued monitoring. Although there was no postoperative sore throat or hoarseness of voice, there was suspicion of pulmonary congestion on chest radiography. On arterial blood gas analysis, the partial pressure of oxygen (PaO_2_) while breathing room air was 64 mmHg. However, the patient was not dyspneic, and pulmonary congestion and PaO_2_ improved over time. On postoperative day 2, the patient was transferred to the general ward without any significant problems.

## Discussion and conclusions

In the present case, conventional direct laryngoscopy was difficult for several reasons. First, the patient could not assume the supine position because of thoracolumbar fusion due to spondylitis. Therefore, he was placed in a 30–40 degree head-up position, even with a full neuromuscular blockade. Second, the atlanto-occipital joint was in the fully extended position due to severe kyphosis, limiting further extension. Hence, bag-mask ventilation was also performed using a two-handed technique while standing in front of the patient. Third, because of the sitting position during intubation, the oro-pharyngeal axis of the patient and the line of vision of the anesthetist were not aligned correctly; this would have, nevertheless, been the case even if a video laryngoscope was used.

Given these difficulties, we elected to use a face-to-face intubation technique involving a lightwand. Face-to-face intubation, also known as the “Tomahawk” or “Pickaxe” method, is more successful when performed by two operators. In the face-to-face intubation technique, the laryngoscope is held in the right hand, blade directed upward, with the patient in the upright or sitting position. After opening the airway, the curved blade of the laryngoscope is positioned in the left of the patient’s mouth. The blade is slid down over the tongue along the left side while pushing it to the right, down to the level of the epiglottis. Following this, the second operator inserts the tracheal tube to the desired depth [4]. The face-to-face intubation technique can be used with other intubation devices, including the laryngeal mask airway, the video laryngoscope, and the direct laryngoscope [5, 6].

Face-to-face intubation in the sitting position has several advantages compared with the conventional intubation technique. First, in a sitting position, gravity facilitates the downward movement of the soft tissues of the neck, making it easier to intubate [7]. Furthermore, without gravitational fixation of the head to the bed, the head and neck may be more mobile in a sitting position, making it easier to align the oro-pharyngeal-tracheal axis [7]. Second, in obese patients, the sitting position leads to downward gravitational movement of the chest, neck, and submandibular region, facilitating airway management. In addition, in obese patients, the time to desaturation can be significantly increased if the patient is pre-oxygenated in a sitting position compared with the supine position [8]. Finally, there are several disease states in which the sitting position is more favorable, including patients with active oral bleeding; the sitting position is also more optimal in respiratory distress due to epiglottitis, asthma, chronic obstructive pulmonary disease, and congestive heart failure, by improving respiratory mechanics. In addition, patients with a full stomach may also benefit from the sitting position [7, 9].

In the present case and unlike previously reported, it is noteworthy that we performed successful face-to-face intubation using a lightwand. Face-to-face lightwand intubation may have advantages compared with other intubation devices. First, it can be easily performed under lighted stylet guidance, even if there is only one operator, compared with previously reported face-to-face intubation techniques for which two operators have been recommended [4, 10]. Furthermore, use of the lightwand is associated with a similar success rate on the first intubation attempt and in a shorter period of time in patients with anticipated difficult airway, compared with the video laryngoscope [11, 12]. Second, using a lightwand may be less traumatic compared with other intubation devices because it enters along the base of the tongue and may cause less damage to the pharyngeal wall or soft tissue [11]. After lightwand intubation, upper airway traumas, including bleeding, sore throat, hoarseness, and dysphagia, were minor in nature [10]. Third, lightwand intubation tends to cause less hemodynamic instability compared with direct laryngoscopy [10, 12]. Finally, unlike the fiberoptic bronchoscope with high maintenance costs, the lightwand is efficient, easy to manage, and is relatively inexpensive [13, 14]. In fact, in most cases of anticipated difficult intubation, awake intubation using fiberoptic bronchoscopy can be considered; however, there may also be limitations in that both an available device and an experienced anesthetist are required. [15]. In the present case, we considered fiberoptic bronchoscopy as the first alternative method, but it was temporarily unavailable. However, the patient was well ventilated with bimanual mask holding in face-to-face position, thereby making it possible to opt for face-to-face lightwand intubation; otherwise, one should wait for fiberoptic bronchoscopy to become available or for the the patient to wake up.

Patients with a severe vertebral column anomaly may not present a normal oro-pharyngeal-tracheal axis. Hence, it would be better to use a two-handed face mask technique in the face-to-face position, with ventilation performed by an assistant or a mechanical ventilator. When fiberoptic bronchoscopy is unavailable, a face-to-face lightwand intubation technique may be a useful alternative, even with a single operator, for successful tracheal intubation in patients who cannot lie supine.

## Abbreviations

FEV_1_: Forced expiratory volume in 1 s
FVC: Forced vital capacity
PaO_2_: Partial pressure of arterial oxygen
